# Bacterial profile, antibiotic susceptibility pattern and associated factors among pregnant women with Urinary Tract Infection in Goba and Sinana Woredas, Bale Zone, Southeast Ethiopia

**DOI:** 10.1186/s13104-018-3910-8

**Published:** 2018-11-08

**Authors:** Solomon Taye, Motuma Getachew, Zelalem Desalegn, Abera Biratu, Khan Mubashir

**Affiliations:** 1 Microbiology, Immunology and Parasitology Department, College of Medicine and Health Sciences, Wachemo University, P.O. Box 667, Hossana, Ethiopia; 2 College of Medicine and Health Sciences, Wollega University, P.O. Box 395, Nekemte, Ethiopia; 3 Microbiology, Immunology and Parasitology Department, School of Medicine, Addis Ababa University, P.O. Box 9086, Addis Ababa, Ethiopia; 4 Department of Public Health, College of Medicine and Health Sciences, Madda Walabu University, P.O. Box 302, Bale Goba, Ethiopia; 5 Department of Biochemistry, School of Medicine, Goba Referral Hospital, Madda Walabu University, P.O. Box 302, Bale Robe, Ethiopia

**Keywords:** Urinary tract infection, Bacteriuria, Pregnant women, Antibiotic susceptibility, Antibiotic resistance

## Abstract

**Objective:**

Urinary tract infection (UTI) is one of the commonest infections affecting millions worldwide, especially pregnant women. It can lead to poor maternal and perinatal outcomes. Untreated UTI can be associated with serious obstetric complications. So the objective of present study was to determine the bacterial profile, antibiotic susceptibility pattern and associated factors of UTI among pregnant women in Goba and Sinana Woredas, Bale Zone, Southeast Ethiopia.

**Results:**

The overall prevalence of UTI was 44/169 (26%) with 18/51 (35.3%) in symptomatic and 26/118 (22%) in asymptomatic pregnant women, respectively. Of the 44 bacterial isolates, *E. coli* 12/44 (27.3%), *K. pneumonia* 9/44 (20.5%) and *S. marcescens* 4/44 (9.1%) were the commonest bacterial pathogens. *C. freundii* 3/44 (6.8%)*, M. morganii* 3/44 (6.8%)*, P. aeruginosa* 3/44 (6.8%) and *S. enteritidis* 3/44 (6.8%) isolates were the moderately identified bacterial species. *K. oxytoca* 1/44 (2.3%) was the least common bacterium to be detected. The antibiotic susceptibility pattern showed that 90.9%, 88.6% and 86.3% of the isolates were sensitive to amoxicillin/clavulanic acid, gentamycin and norfloxacin, respectively. Significant bacteriuria was associated with low educational status (p = 0.024; AOR = 6.617; CI = 1.87–9.94) and kidney problems (p = 0.018; AOR = 0.286; CI = 1.19–2.81).

**Electronic supplementary material:**

The online version of this article (10.1186/s13104-018-3910-8) contains supplementary material, which is available to authorized users.

## Introduction

In women UTIs account for about 25% of all infections thus being one of the most frequent clinical bacterial infections [1]. During pregnancy there occur many anatomical and hormonal changes in women which make them susceptible to develop UTI. Around 20% of the pregnant women are reported to have UTI and it is the most common cause for admission to obstetric wards [2]. Its occurrence usually starts in week 6 and becomes most frequent during weeks 22–24 of pregnancy. There are many factors responsible like dilatation of urethra, increased bladder volume and decreased bladder tone, along with decreased urethral tone which contributes to increased urinary stasis and uretero-vesical reflux. In addition, up to 70% of women during pregnancy have glucose in urine, which increases the chances of bacterial growth in the urine [3]. Untreated UTI in pregnant women may have serious consequences like intrauterine growth restriction, preeclampsia, caesarean delivery and preterm deliveries [4]. Further untreated asymptomatic bacteriuria (ASB) is a significant risk of acute pyelonephritis in later pregnancy [5].

Urinary tract infection is considered one of the most common medical complications during pregnancy [6]. During pregnancy a number of risk factors of UTI are reported depending on the social, biological and geographical settings [7]. The commonest cause of UTI among pregnant women has been found to be *E. coli* because of its multidrug resistant strains [8]. Since ASB and obvious UTI has a close association, screening and treatment of pregnant women with ASB may also help to reduce adverse outcome for the child such as pre-term labour and low birth weight [9]. However, antibiotic resistance of urinary tract pathogens has been increasing worldwide, especially to the commonly used antimicrobials [10] and pattern of antibiotic resistance in a wide variety of pathogenic organisms may vary over short periods and depend on site of isolation and different environmental conditions [11].

Data on the current distribution and antibiotic drug susceptibility patterns among urinary bacterial isolates from Ethiopian pregnant women patients; particularly in the study area is limited. Therefore, this study was aimed to determine the bacterial profile, antibiotic susceptibility pattern and associated factors of UTI among pregnant women visiting health institutions in Goba and Sinana Woredas, Bale Zone, Southeast Ethiopia.

## Main text

### Materials and methods

#### Study design, population and sampling technique

The study was conducted in Bale Zone, Southeast Ethiopia from January to April 2014 at different health institutions in Goba and Sinana Woredas. Institution based cross-sectional study design was employed on pregnant women visiting health institutions for ante-natal care. Six health institutions (Goba and Robe town hospitals, Goba health center, Ititu Sura health center, Sanbitu health center and Kebira Temo health post) were selected using lottery method. The sample size was determined using the single proportion population formula. For the current study 169 pregnant women were selected using systematic random sampling method. Pregnant women who were on antibiotic treatment during sample collection were excluded. Pre-structured questionnaires prepared were used to collect data on socio-demographic and associated factors.

#### Urine specimen collection and bacteriological investigation

One hundred sixty-nine (169) early morning midstream urine specimens were collected using leak proof sterile plastic containers. Using calibrated wire loop (0.001 ml) samples were inoculated on to cysteine lactose electrolyte deficient medium (CLED, Oxoid) and mannitol salt agar (MSA, Oxoid). After incubation at 37 °C for 18–24 h colonies were counted to check significant growth. Colony counts yielding bacterial growth of 10^5^ colony forming unit (CFU)/ml were regarded as significant for bacteriuria. Colonies from CLED were subcultured into MacConkey agar (Oxoid) and blood agar plates (BAP, Oxoid) and incubated at 37 °C for 18–24 h. Bacteria were identified using colony characteristics, gram reaction of the organisms and biochemical tests following standard procedure. All procedures were conducted in Biomedical Research Laboratory of College of Medicine and Health Science, Madawalabu University. Culture media was tested for sterility and performance. Standard strains of *E. coli* (ATCC 25922), *S. aureus* (ATCC 25923) and *P. aeruginosa* (ATCC 27853) were used as reference strains for culture and sensitivity testing.

#### Antibiotic susceptibility testing

Antibiotic susceptibility testing was performed on all significant isolates according to the criteria of Clinical and Laboratory Standards Institute (CLSI) [12]. Agar disc diffusion method was used to determine susceptibility of the isolates. Individual colonies were suspended in 5 ml normal saline to 0.5 McFarland standard in order to standardize the inoculum size and using sterile swabs the suspensions were evenly distributed over the entire surface of Muller Hinton agar. Using a sterile forceps the antibiotic discs were placed on the inoculated plates and incubated at 37 °C for 18–24 h. The drug discs (Oxoid) tested were ampicillin (10 μg), amoxicillin/clavulanic acid (20/10 μg), ceftazidime (30 μg), cefotaxime (30 μg), doxycycline (30 μg), gentamycin (10 μg), nitrofurantoin (300 μg), norfloxacin (10 μg) and tetracycline (30 μg). Diameter of the zone of inhibition around the disc was measured using a digital metal caliper and isolates were classified as sensitive, intermediate and resistant according to CLSI [14]. Symptomatic pregnant women were given amoxicillin/clavulanic acid(250/125 mg/TID) as empirical treatment before culture results.

#### Data analysis

Data were checked for completeness and entered into EPI Info and transferred to SPSS version 16. The base line characteristics of the study population were summarized using frequencies, mean and standard deviation. Internal comparison was made using binary logistic regression to determine the independent effect of the variables by calculating the strength of the association between UTI and associated factors using odds ratio (OR) and 95% confidence interval (CI). Adjusted OR (for variables which were statistically significant in binary logistic analysis) was computed using multivariable logistic regression to control the confounding variables. A P-value of < 0.05 was considered as an indicator of statistical significance.

#### Ethical issue

The research was ethically cleared and approved by MaddaWalabu University ethical review committee and a letter was given with reference number 1093-MWU/EC. Written informed consent was obtained from all study participants for participation and publication of the research. For each confirmed infection case, the responsible clinician of the participant was contacted and treatment was given as per the culture result and antibiotic susceptibility pattern.

### Results

#### Socio-demographic characteristics of study participants

Of total study population one hundred thirty (76.9%) lived in urban areas. More than half (85/169 (50.3%) of study subjects were from Robe hospital followed by 64/169 (37.9%) from Goba hospital. Most of the participants were in the age group of 18–27 years (76.3%) and 28–37 years (20.7%), respectively. 98.2% of the participants were married and 1.8% were divorced (Table 1).

**Table 1:** Socio-demographic characteristics of pregnant women (n = 169) visiting health institutions in Goba and Sinana Woredas, Southeast Ethiopia, 2014

Characteristics	Total n (%)	Urinary tract infection		Crude OR (95% CI)	P value
		n (%) positive for UTI	n (%) negative for UTI		
Health institution					
Goba Hospital	64 (37.9)	14 (21.9)	50 (78.1)	0.64 [0.30–1.35]	0.914
Robe Hospital	85 (50.3)	26 (30.6)	59 (69.4)	1.14 [0.10–13.10]	
Goba Health Center	2 (1.2)	0 (0.0)	2 (100)	0.97 [0.23–4.10]	
Ititu Sura Health Center	3 (1.8)	1 (33.3)	2 (66.7)	0.01 [0.32–1.21]	
Sanbitu Health Center	5 (2.9)	0 (0.0)	5 (100)	0.05 [0.83–1.95]	
Kebira Temo Health post	10 (5.9)	3 (30)	7 (70)	1	
Residence					
Urban	130 (76.9)	34 (26.2)	96 (73.8)	0.97 [ 0.43–2.21]	0.949
Rural	39 (23.1)	10 (25.6)	29 (74.4)	1	
Age interval in years					
18–27	129 (76.3)	31 (31.8)	98 (33.2)	5.11 [0.87–2.69]	0.281
28–37	35 (20.7)	10 (59.8)	25 (57.9)	6.46 [0.76–3.18]	
38–47	5 (3)	3 (60)	2 (40)	1	
Marital status					
Married	166 (98.2)	44 (26.5)	122 (73.5)	0.01 [0.11–1.25]	0.999
Divorced	3 (1.8)	0 (0.0)	3 (100)	1	
Educational status					
Illiterate	25 (14.8)	8 (18.2)	17 (10.1)	1.06 [1.012–3.937]	0.044
Read and write	10 (5.9)	6 (3.6)	4 (2.4)	0.99 [0.37–2.67]	
Elementary (1–8)	63 (37.3)	20 (11.8)	43 (25.4)	0.47 [0.15–1.41]	
High school (9–12)	50 (29.6)	9 (5.3)	41 (24.3)	0.11 [0.01–0.94]	
12+	21 (12.4)	1 (0.6)	20 (11.8)	1	
Religion					
Orthodox Christian	82 (48.5)	18 (22)	64 (78)	4.54 [0.33–3.96]	0.492
Muslim	79 (46.7)	26 (32.9)	53 (67.1)	7.93 [0.10–6.19]	
Protestant	8 (4.8)	0 (3.0)	8 (3.0)	1	
Occupation					
House wife	128 (75.7)	35 (27.3)	93 (72.7)	0.57 [0.15–2.10]	0.925
Government employee	17 (10.1)	3 (17.6)	14 (82.4)	0.66 [0.07–6.15]	
Private employee	5 (2.9)	1 (20)	4 (80)	1.33 [0.38–4.69]	
Merchant	12 (7.1)	4 (33.3)	8 (66.7)	0.53 [0.06–4.71]	
Farmer	6 (3.6)	1 (16.7)	5 (83.3)	0.01 [0.11–0.99]	
Student	1 (0.6)	0 (0.0)	1 (100)	1	

#### Prevalence of urinary tract infection

The overall prevalence of UTI among the study participants was 44/169 (26%). Furthermore, 18/51 (35.3%) of them were symptomatic and 26/118 (22%) were asymptomatic. *E. coli* 12/44 (27.3%), *K. pneumonia* 9/44 (20.5%) and *S. marcescens* 4/44 (9.1%) were the commonest bacterial uropathogens. *C. freundii* 3/44 (6.8%)*, M. morganii* 3/44 (6.8%)*, P. aeruginosa* 3/44 (6.8%) and *S. enteritidis* 3/44 (6.8%) isolates were the moderately identified bacterial species. *K. oxytoca* 1/44 (2.3%) was the least detected bacterium. There were no Gram-positive bacteria detected and none of the isolates were found in mixed growth (Additional file 1: Figure S1). Of the infected pregnant women, 26/44 (59.1%) were from Robe hospital and 14/44 (31.8%) of them were attendants of Goba hospital. In this study, 34/44 (77.3%) of UTI positive subjects were urban dwellers. And 31/44 (70.5%) of UTI cases were under the age of 27 years (Table 1).

#### Factors associated with urinary tract infection

Significant bacteriuria was associated with low educational status (p = 0.044; COR = 1.06; CI = 1.012–3.937) and those pregnant women with kidney problems (p = 0.030; COR = 2.77; CI = 1.61–7.97). All the remaining socio-demographic characteristics and associated factors were non-significantly associated with UTI (Tables 1, 2). After adjusting for those variables which were statistically significant in binary logistic analysis, multivariable logistic analysis was conducted. Low educational status (illiteracy) (p = 0.024; AOR = 6.617; CI = 1.87–9.94) and kidney problems (p = 0.018; AOR = 0.286; CI = 1.19–2.81) were significantly associated with UTI (Table 3).

**Table 2:** Associated factors of UTI among pregnant women (n = 169) visiting health institutions in Goba and Sinana Woredas, Southeast Ethiopia, 2014

Associated factors	Total n (%)	Urinary tract infection			
		Positive no (%)	Negative no (%)	Crude OR (95% CI)	P value
Gravidity					
1st	61 (36.1)	16 (26.2)	45 (73.8)	0.74 [0.31–1.77]	0.346
2nd	53 (31.4)	11 (20.8)	42 (79.2)	0.86 [0.31–2.38]	
3rd	30 (17.8)	7 (23.3)	23 (76.7)	0.88 [0.70–5.01]	
≥ 4	25 (14.8)	10 (40)	15 (60)	1	
Gestational age					
1st trimester	8 (4.7)	3 (37.5)	5 (62.5)	0.54 [0.11–2.63]	0.743
2nd trimester	45 (26.6)	11 (24.4)	34 (75.6)	0.58 [0.13–2.58]	
3rd trimester	116 (68.7)	30 (25.9)	86 (74.1)	1	
Diabetes status					
No	159 (94.1)	42 (26.4)	117 (73.6)	0.70 [0.14–3.41]	0.655
DK	10 (5.9)	2 (20)	8 (80)	1	
Hypertension status					
Yes	4 (2.4)	0 (0.0)	4 (100)	5.84 [0.62–7.99]	0.966
No	162 (95.9)	43 (26.5)	119 (73.5)	8.10 [0.87–15.09]	
DK	3 (1.8)	1 (33.3)	2 (66.7)	1	
Kidney problem					
Yes	22 (13)	10 (45.5)	12 (54.5)	2.77 [1.61–7.97]	0.030
No	147 (87)	34 (23.1)	113 (76.9)	1	
Gynecological surgery					
Yes	6 (3.6)	0 (0.0)	6 (100)	5.97 [0.23–8.19]	0.999
No	163 (96.4)	44 (27)	119 (73)	1	
Current tooth or mouth problem					
Yes	24 (14.2)	5 (20.8)	19 (79.2)	1.40 [0.49–4.00]	0.532
No	145 (85.8)	39 (26.9)	106 (73.1)	1	
At least two symptoms					
Yes	51 (30.2)	18 (35.3)	33 (64.7)	0.52 [1.25–1.97]	0.074
No	118 (69.8)	26 (22)	92 (78)	1	
Prolonged antibiotic therapy					
Yes	12 (7.1)	2 (16.7)	10 (83.3)	1.83 [0.38–8.68]	0.449
No	157 (92.9)	42 (26.8)	115 (73.2)	1	
Urinary catheterization					
Yes	2 (1.2)	0 (0.0)	2 (100)	5.78 [1.14–9.89]	0.999
No	167 (98.8)	44 (26.3)	123 (73.7)	1	
HIV status					
Yes	9 (5.3)	2 (22.2)	7 (77.8)	1.22 [0.24–6.12]	0.853
No	151 (89.4)	39 (25.8)	112 (74.2)	1.75 [0.22–14.22]	
DK	9 (5.3)	3 (33.3)	6 (46.7)	1	
STI					
Yes	7 (4.1)	2 (28.6)	5 (71.4)	0.82 [0.15–4.40]	0.309
No	154 (91.3)	38 (24.7)	116 (75.3)	2.50 [0.29–21.40]	
DK	8 (4.7)	4 (50)	4 (50)	1	

*DK* don’t know

**Table 3:** Crude and adjusted OR of associated factors of UTI among pregnant women (n = 169) visiting health institutions in Goba and Sinana Woredas, Southeast Ethiopia, 2014

Associated factors	Urinary tract infection					
	Positive n (%)	Negative n (%)	Crude OR (95% CI)	P value	Adjusted OR	P value
Educational status						
Illiterate	8 (68.2)	17 (64.8)	1.06 [1.012–3.937]	0.044	6.617 [1.87–9.94]	0.024
Read and write	6 (31.8)	4 (35.2)	0.99 [0.37–2.67]		1.35 [043–4.25]	
Elementary (1–8)	20 (69.0)	43 (69.0)	0.47 [0.15–1.41]		0.53 [0.15–1.92]	
High school (9–12)	9 (69.0)	41 (69.0)	0.11 [0.01–0.94]		0.17 [0.02–1.74]	
12+	1 (69.0)	20 (69.0)	1		1	
Kidney problem						
Yes	10 (45.5)	12 (54.5)	2.77 [1.61–7.97]	0.030	0.286 [1.19–2.81]	0.018
No	34 (23.1)	113 (76.9)	1		1	

#### Antibiotic susceptibility test pattern

The antibiotic susceptibility test pattern showed that 90.9%, 88.6% and 86.3% of the isolates were sensitive to amoxicillin/clavulanic acid, gentamycin and norfloxacin, respectively. The isolates were least sensitive to cefotaxime (18%), ceftazidime (19%), doxycycline (22%) and tetracycline (23%). None of the isolates were resistant to amoxicillin/clavulanic acid. Except amoxicillin/clavulanic acid, *E. coli* had showed resistance to all tested drugs at least in one pregnant woman. Six *E. coli* isolates were resistant to ampicillin and doxycycline. Furthermore, eight *E. coli* isolates were also resistant to cefotaxime, tetracycline, norfloxacin and nitrofurantoin. Conversely, *P. vulgaris* was the isolate to show the least resistance against the tested drugs. Almost all isolates showed intermediate level of resistance against ceftazidime, cefotaxime and tetracycline (36.4%). Low level of resistance (15%) was observed against gentamycin and norfloxacin (Additional file 2: Table S1).

### Discussion

This study assessed the bacterial profile, antibiotic susceptibility pattern and associated factors of UTI among pregnant women visiting health institutions in Goba and Sinana Woredas, Southeast Ethiopia. The prevalence of UTI 44/169 (26%) in this study was higher than the studies conducted in Addis Ababa Ethiopia 11.6% [13], Bahirdar Ethiopia 9.5% [14], Gondar Ethiopia 10.4% [15] and Sudan 12.1% [16]. However this value was lower than prevalence of UTI among pregnant women in Pakistan 28.8% [17]. This variation may be because of differences in the environment, social habits, standard of personal hygiene or may be due to the low economic status of the study subjects. The differences in design and methodologies might also affect comparison of prevalence in different surveys [14]. The prevalence of bacteriuria among symptomatic and asymptomatic pregnant women in our study were 18/51 (35.3%) and 26/118 (22%), respectively which was higher than studies done in Bahirdar (8.5% and 18.9%) [17], Gondar (15.9% and 10.2%) [14] and Sudan (12.1% and 14.7%) [16].

The most prevalent uropathogen identified was *E. coli* (27.3%) followed by *K. pneumoniae* 9/44 (20.5%) and *S. marcescens* 4/44 (9.1%). These uropathogens were also identified in other similar studies in Addis Ababa Ethiopia 44% [13], Bahirdar Ethiopia 45.7% [14], Gondar Ethiopia 47.5% [15] and Sudan 42.4% [16]. The major contributing factor for isolating higher *E. coli* is due to urine stasis in pregnancy which favors for *E. coli* strain colonization [14, 18]. *K. pneumoniae* is commonly found in wet areas especially in health institutions and can easily infect pregnant women [19]. Another reason could be due to poor genital hygienic practices by pregnant women who may find it difficult to clean properly after defecating or clean their genital after passing urine during their pregnancy [20]. The differences in prevalence rate between the study areas might be due to culture, practice, living standards and category of the study population in addition to the period of study and the methods employed for urine examination [19, 20].

Most isolates were sensitive to amoxicillin/clavulanic acid, gentamycin and norfloxacin which is in accordance with previous studies done in Gondar, Addis Ababa and Bahirdar Ethiopia [13–15] and Sudan [16]. However, the observed drug resistance pattern in our study (36.4%) was very much lower than study carried in Bahirdar (93.1%) [20]. *E. coli* with its multidrug resistance pattern in the present study is in conformity with other reports in Pakistan and Singapore [21, 22]. Inappropriate antimicrobial use due to the lack of adequate knowledge about drugs can lead to inadequate therapy and contribute to further drug resistance [8, 19].

Significant bacteriuria was associated with low educational status (p = 0.024; AOR = 6.617; CI = 1.87–9.94) and kidney problems (p = 0.018; AOR = 0.286; CI = 1.19–2.81). This may be attributed to the limited knowledge of pregnant women about the transmission and prevention of uropathogens. Furthermore, physician confirmed kidney problems like kidney stones, glomerulonephritis, genetic abnormality of kidneys, defects of adrenal gland and renal anomalies are the major predisposing factors for pregnant women to develop UTI [23].

## Limitations

This is a short period study and involved those women only who attended the health care system. In order to validate these results the time period and area of this study has to be increased and the population should include women from the community.

## Additional files

**Additional file 1: Figure S1.** Prevalence of isolated bacteria among pregnant women (n = 169) visiting health institutions in Goba and Sinana Woredas, Southeast Ethiopia, 2014.

**Additional file 2: Table S1.** Antibiotic susceptibility pattern of bacterial isolates from pregnant women (n = 169) visiting health institutions in Goba and Sinana Woredas, Southeast Ethiopia, 2014.

## Abbreviations

AOR: adjusted odds ratio
ASB: asymptomatic bacteriuria
ATCC: American type culture collection
BAP: blood agar plate
*C. freundii*: *Citrobacter freundii*
CFU: colony forming unit
CI: confidence intervals
CLED: cysteine lactose electrolyte deficient medium
CLSI: Clinical and Laboratory Standards Institute
COR: crude odds ratio
*E. aerogens*: *Enterobacter* aerogenes
EPI Info: epidemiological information
*E. coli*: *Escherichia coli*
*K. pneumonia*: *Klebsiella pneumonia*
*K. oxytocae*: *Klebsiella oxytoca*
*M. morganii*: *Morganella morganii*
MSA: mannitol salt agar
*P. vulgaris*: *Proteus vulgaris*
*P. aeruginosa*: *Psuedomonas aeruginosa*
*S. enteritidis*: *Salmonella enteritidis*
*S*. *marcescens*: *Serratia marcescens*
STIs: sexually transmitted infections
*S. aureus*: *Staphylococcus aureus*
SPSS: Statistical package for social Sciences
SB: symptomatic bacteriuria
UTI: urinary tract infection
