# Supplementary material for: Bacterial profile, antibiotic susceptibility pattern and associated factors among pregnant women with Urinary Tract Infection in Goba and Sinana Woredas, Bale Zone, Southeast Ethiopia
Source: BMC Res Notes. 2018 Nov 8;11:799. doi: 10.1186/s13104-018-3910-8 (PMC6225670; doi:10.1186/s13104-018-3910-8)
Supplement: Supplementary file 1 — Additional file 1: Figure S1. Prevalence of isolated bacteria among pregnant women (n = 169) visiting health institutions in Goba and Sinana Woredas, Southeast Ethiopia, 2014. [file 13104_2018_3910_MOESM1_ESM.pdf]

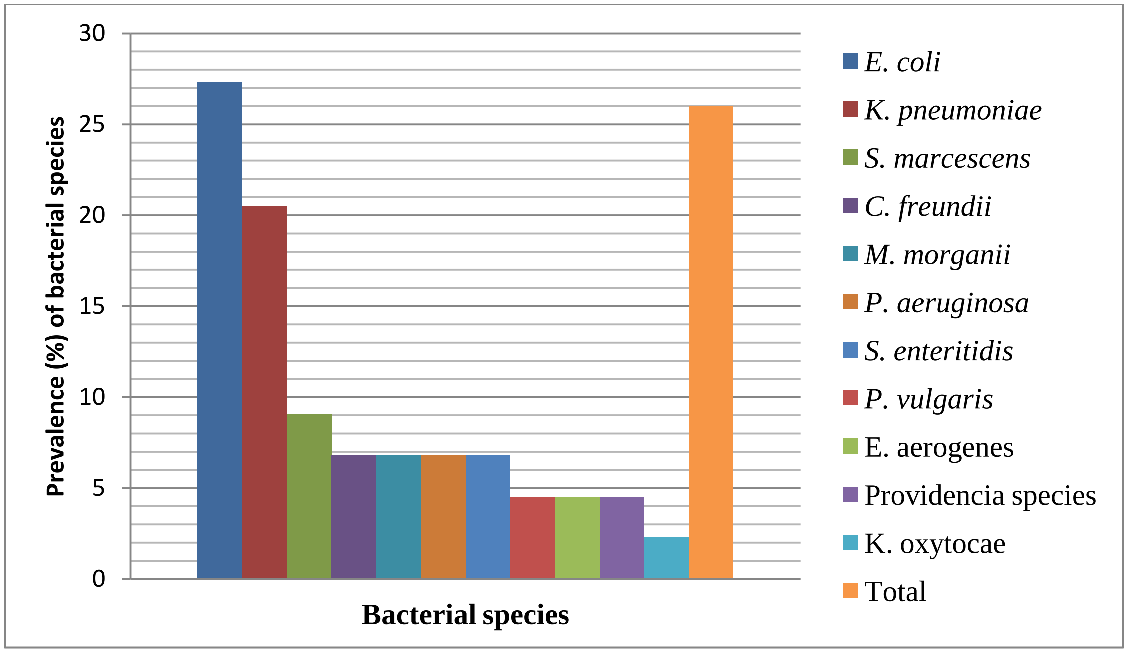

Figure S1: Prevalence of isolated bacteria among pregnant women (n=169) visiting health institutions in Goba and Sinana Woredas, Southeast Ethiopia, 2014.
