# Supplementary material for: Bacterial profile, antibiotic susceptibility pattern and associated factors among pregnant women with Urinary Tract Infection in Goba and Sinana Woredas, Bale Zone, Southeast Ethiopia
Source: BMC Res Notes. 2018 Nov 8;11:799. doi: 10.1186/s13104-018-3910-8 (PMC6225670; doi:10.1186/s13104-018-3910-8)
Supplement: Supplementary file 2 — Additional file 2: Table S1. Antibiotic susceptibility pattern of bacterial isolates from pregnant women (n = 169) visiting health institutions in Goba and Sinana Woredas, Southeast Ethiopia, 2014. [file 13104_2018_3910_MOESM2_ESM.pdf]

Table S1: Antibiotic susceptibility pattern of bacterial isolates from pregnant women (n=169) visiting health institutions in Goba and Sinana Woredas, Southeast Ethiopia, 2014.

Bacterial Species	Total n(%)	S/I/R			Antibiotic Discs Tested						
			AMP n(%)	AMC n(%)	CAZ n(%)	CTX n(%)	DO n(%)	CN n(%)	F n(%)	NOR n(%)	TE n(%)
E. coli	12 (7.1)	S	9 (75)	10 (83.3)	7 (58.3)	7 (58.3)	5 (41.7)	9 (75)	10 (83.3)	8 (66.7)	7 (58.3)
		I	-	2 (16.7)	4 (33.3)	3 (25)	4 (33.3)	2 (16.7)	-	2(16.7)	3 (25)
		R	3 (25)	-	1 (8.4)	2 (16.7)	3 (25)	1 (8.4)	2 (16.7)	2 (16.7)	2 (16.7)
K. pneumoniae	9 (5.3)	S	6 (66.7)	8 (88.9)	4 (44.5)	3 (33.3)	4 (44.5)	9 (100)	7 (77.8)	9 (100)	5 (55.6)
		I	-	1 (11.1)	2 (22.2)	1 (11.1)	-	-	2 (22.2)	-	-
		R	3 (33.3)	-	3 (33.3)	5 (55.6)	5 (55.6)	-	-	-	4 (44.5)
S. marcescens	4 (2.4)	S	2 (50)	4 (100)	1 (25)	-	3 (75)	4 (100)	1 (25)	4 (100)	3 (75)
		I	-	-	-	2 (50)	1 (25)	-	2 (50)	-	-
		R	2 (50)	-	3 (75)	2 (50)	-	-	1 (25)	-	1 (25)
C. freundii	3 (1.8)	S	3 (100)	3 (100)	1 (33.3)	2 (66.7)	2 (66.7)	2 (66.7)	3 (100)	2 (66.7)	1 (33.3)
		I	-	-	1 (33.3)	1 (33.3)	-	-	-	-	1 (33.3)
		R	-	-	1 (33.3)	-	1 (33.3)	1 (33.3)	-	1 (33.3)	1 (33.3)
M. morganii	3 (1.8)	S	3 (100)	3 (100)	1 (33.3)	1 (33.3)	2 (66.7)	3 (100)	2 (66.7)	3 (100)	1 (33.3)
		I	-	-	-	1 (33.3)	1 (33.3)	-	1 (33.3)	-	-
		R	-	-	2 (66.7)	1 (33.3)	-	-	-	-	2 (66.7)
P. aeruginosa	3 (1.8)	S	3 (100)	3 (100)	-	-	2 (100)	3 (100)	3 (100)	2 (66.7)	1 (33.3)
		I	-	-	-	-	1 (100)	-	-	-	-
		R	-	-	3 (100)	3 (100)	-	-	-	1 (33.3)	2 (66.7)
S. enteritidis	3 (1.8)	S	2 (66.7)	3 (100)	-	2 (66.7)	3 (100)	3 (100)	1 (33.3)	3 (100)	2 (66.7)
		I	-	-	2 (66.7)	-	-	-	-	-	-
		R	1 (33.3)	-	1 (33.3)	1 (33.3)	-	-	2 (66.7)	-	1 (33.3)

P. vulgaris	2 (1.2)	S	2 (100)	2 (100)	2 (100)	-	-	2 (100)	-	2 (100)	2 (100)
		I	-	-	-	1 (50)	1 (50)	-	1 (50)	-	-
		R	-	-	-	1 (50)	1 (50)	-	1 (50)	-	-
E. aerogenes	2 (1.2)	S	1 (50)	1 (50)	1 (50)	1 (50)	-	2 (100)	2 (100)	2 (100)	-
		I	-	1 (50)	-	1 (50)	-	-	-	-	-
		R	1 (50)	-	1 (50)	-	2 (100)	-	-	-	2 (100)
Providencia species	2 (1.2)	S	1 (50)	2 (100)	1 (50)	1 (50)	-	2 (100)	2 (100)	2 (100)	1 (50)
		I	-	-	-	-	2(100)	-	-	-	-
		R	1 (50)	-	1 (50)	1 (50)	-	-	-	-	1 (50)
K. oxytocae	1 (0.6)	S	1 (100)	1 (100)	1 (100)	1 (100)	1 (100)	-	-	1 (100)	-
		I	-	-	-	-	-	1 (100)	1 (100)	-	1 (100)
		R	-	-	-	-	-	-	-	-	-
**Total**	**44 (26)**	S	**33 (75)**	**40 (90.9)**	**19 (43.2)**	**18 (40.9)**	**22 (50)**	**39 (88.6)**	**31 (70.5)**	**38 (86.3)**	**23 (52.3)**
		I	**-**	**4 (9.1)**	**9 (20.4)**	**10 (22.7)**	**12 (27.3)**	**3 (6.8)**	**7 (15.9)**	**4 (9.1)**	**5 (11.3)**
		R	**11 (25)**	**-**	**16 (36.4)**	**16 (36.4)**	**10 (22.7)**	**2 (4.6)**	**6 (13.6)**	**2 (4.6)**	**16 (36.4)**

AMP=Ampicillin; AMC=amoxicillin/clavulanic acid; CAZ=Ceftazidime; CTX=Cefotaxime; DO=Doxycycline; CN=Gentamyicn; F=Nitrofurantoin; NOR=Norfloxacin; TE=Tetracycline; S=sensitive; I=intermediate; R=resistant
